# Maternal Autoimmune Disease and Childhood‐Onset Type 1 Diabetes: A Nationwide Population‐Based Nested Case‐Control Study

**DOI:** 10.1155/pedi/3418021

**Published:** 2026-01-20

**Authors:** Hsin-Chien Yen, Ching-Heng Lin, Ming-Chih Lin

**Affiliations:** ^1^ Department of Post-Baccalaureate Medicine, College of Medicine, National Chung Hsing University, Taichung, Taiwan, nchu.edu.tw; ^2^ Division of Neonatology, Children’s Medical Center, Taichung Veterans General Hospital, Taichung, Taiwan, vghtc.gov.tw; ^3^ Doctoral Program in Translational Medicine, National Chung Hsing University, Taichung, Taiwan, nchu.edu.tw; ^4^ Department of Medical Research, Taichung Veterans General Hospital, Taichung, Taiwan, vghtc.gov.tw; ^5^ Institute of Public Health and Community Medicine Research Center, National Yang Ming Chiao Tung University, Taipei, Taiwan, nctu.edu.tw; ^6^ Department of Epidemiology and Public Health, UCL, London, UK, ucl.ac.uk; ^7^ School of Medicine, National Yang Ming Chiao Tung University, Taipei, Taiwan, nctu.edu.tw; ^8^ School of Medicine, Chung Shan Medical University, Taichung, Taiwan, csmu.edu.tw

**Keywords:** autoimmune thyroiditis, maternal autoimmune disease, rheumatoid arthritis, type 1 diabetes mellitus (T1DM)

## Abstract

**Introduction:**

Type 1 diabetes mellitus (T1DM) is an autoimmune disease that damages insulin‐producing pancreatic cells, often appearing in childhood. Global incidence is rising at 2%–3% yearly. Its exact cause is unclear. Prenatal exposures and maternal autoimmune disorders have been reported as potential risk factors. This study aimed to explore how maternal autoimmune conditions might correlate with the onset of T1DM, employing a population‐focused approach.

**Methods:**

This is a retrospective population‐based cohort study with a nested case‐control analysis. Primary data were derived from the Maternal and Child Health Database (MCHD) and the National Health Insurance Research Database (NHIRD). This study enrolled a total of 2,036,051 newborns born between 2004 and 2014. They were followed up until the end of 2020. A total of 1273 children under the age of 17 with T1DM were identified from 2004 to 2020. A 1:10 control group, matched by birth date and sex, was selected for comparison. T1DM patients were identified through the Catastrophic Illness Registry Database. Maternal autoimmune diseases were determined using the primary diagnosis codes for hospitalizations and outpatients’ visits.

**Results:**

After adjusting for cofactors, the offspring of mothers with an autoimmune disease had a higher risk of T1DM (adjusted odds ratio [aOR] 1.95, 95% confidence interval [CI]: 1.45–2.63, *p*  < 0.001). For individual autoimmune diseases, T1DM (aOR: 6.81, 95% CI: 2.30–20.16, *p*  < 0.001), Hashimoto thyroiditis (aOR: 3.75, 95% CI: 1.85–7.60, *p*  < 0.001), rheumatoid arthritis (aOR: 2.49, 95% CI: 1.08–5.77, *p* = 0.033), and Graves’ disease (aOR: 1.85, 95% CI: 1.14–2.99, *p* = 0.013) significantly increase the risk of developing T1DM in their children.

**Conclusions:**

Offspring of mothers diagnosed with autoimmune disease, notably T1DM, autoimmune thyroiditis, and rheumatoid arthritis, may indeed have a heightened likelihood of developing T1DM. These findings underscore the importance of targeted screening programs for T1DM in children of affected mothers.

## 1. Introduction

Type 1 diabetes mellitus (T1DM) arises from an autoimmune process that targets and damages the insulin‐producing beta cells located within the islets of Langerhans in the pancreas [1]. The onset of T1DM in childhood displays a pattern where ages at presentation are clustered around two distinct peaks, with one peak typically occurring between 4 and 6 years of age and a second peak emerging during early puberty [2]. The incidence of T1DM is on the rise. There is an annual increase in incidence of ~2%–3% [3]. The development of this condition might result from a complex interplay between various genetic and immunological factors, underscoring its multifaceted nature [4].

The precise underlying mechanisms driving the development of T1DM have yet to be entirely clarified. It has been suggested that it could be initiated by factors like viral infections or other immunological triggers [5, 6]. Periconceptual, prenatal, and early extrauterine environmental stress have a profound impact on lifetime health in offspring, as has been proposed by the developmental origins of health and disease (DOHaD) hypothesis [7]. Thus, an abnormal immune response to specific environmental triggers in individuals with a genetic predisposition may play a role in the development of T1DM [1, 8].

The aim of this study was to investigate the association between mothers with autoimmune diseases and the subsequent development of T1DM in their offspring from a population‐based perspective.

## 2. Methods

### 2.1. Study Design and Data Source

This is a retrospective population‐based cohort study with a nested case–control analysis. The main data source is Taiwan’s National Health Insurance Research Database (NHIRD). Taiwan’s National Health Insurance (NHI) program was launched in 1995. It is a mandatory program that covers 99.7% of Taiwan’s population. In 2002, the NHIRD was established to provide researchers with access to the claims data of NHI beneficiaries. The NHIRD includes each patient’s demographic characteristics, registration, residence, examinations, procedures, diagnoses, prescriptions, surgeries, outpatient services, and inpatient services [9]. In 2015, the Ministry of Health and Welfare (MOHW) expanded the integration of the NHIRD with additional health‐related databases to create Taiwan’s Maternal and Child Health Database (MCHD) at the Health and Welfare Data Center (HWDC) [9]. The MCHD links the National Register of Death (NRD), Birth Certificate Application (BCA), and Taiwan Birth Registry Database (BRD) to the NHIRD. This integration has facilitated a comprehensive linkage between babies’ and mothers’ claims data. Throughout the duration of the study, diagnoses recorded in the NHIRD were classified using the International Classification of Diseases, Ninth and Tenth Revision (ICD‐9 and ICD‐10) formats. To protect patients’ privacy in accordance with confidentiality protocols and to authenticate the validity of the databases, researchers must conduct on‐site analyses at the HWDC through a remote connection to MOHW servers. The study protocol gained approval from the institutional review board of Taichung Veterans General Hospital (CE17178A‐4), which waived the need for written informed consent since all patient data had been anonymized before analysis.

### 2.2. Case Identification

This nested case–control study included all live births in Taiwan between 2004 and 2014. Infants with a diagnosis of congenital anomaly (ICD‐9 code 740–758) were excluded from the analysis. The case group comprised children diagnosed with T1DM, while the control group consisted of children without T1DM. Controls were matched to cases at a 1:10 ratio by birth date and sex. After matching, the same index date, defined as the T1DM diagnosis date of the case, was assigned to the matched controls. The exposure was defined as a maternal autoimmune disease diagnosed prior to delivery, ascertained from medical records before the index date. T1DM was identified via the Catastrophic Illness Registry database in the NHIRD. The National Health Insurance Administration reviews applications for catastrophic illness certification on a case‐by‐case basis. Therefore, all T1DM diagnoses included in the analysis were assumed to be valid. All patients were followed up until the end of 2020.

### 2.3. Maternal Autoimmune Disease and Covariates

Maternal autoimmune diseases were defined as mothers with diagnosis of T1DM (ICD‐9‐CM code 250.01, 250.03, ICD‐10‐CM code E10), Sjogren’s syndrome (ICD‐9‐CM code 710.2, ICD‐10‐CM code M35.0), rheumatoid arthritis (ICD‐9‐CM code 714.0, ICD‐10‐CM code M05‐M06), systemic lupus erythematosus (ICD‐9‐CM code 710.0, ICD‐10‐CM code M32), Hashimoto thyroiditis (ICD‐9‐CM code 245.2, ICD‐10‐CM code E06.3), Graves’ disease (ICD‐9‐CM code 242.0, ICD‐10‐CM code E05.0), dermatomyositis (ICD‐9‐CM code 710.3, ICD‐10‐CM code M33.9), systemic sclerosis (ICD‐9‐CM code 710.1, ICD‐10‐CM code M34.9, M34.8, M34.0, M34.1), celiac disease (ICD‐9‐CM code 579.0, ICD‐10‐CM code K90.0), Addison’s disease (ICD‐9‐CM code 255.4, ICD‐10‐CM code E27.1), or psoriasis (ICD‐9‐CM code 696.0, 696.1, 696.2, 696.8, ICD‐10‐CM code L40.1, L40.2, L40.3, L40.5, L40.8) once during a hospitalization or at least three times for outpatient visits any time prior to delivery (with medical records available from 2000 via the NHIRD, ensuring at least 4–14 years of pre‐delivery data available depending on birth year). We also obtained maternal or neonatal factors that might have had a confounding effect, including gender, gestational age, birth weight, mode of delivery, maternal age, maternal hypertension (ICD‐9‐CM code 401, ICD‐10‐CM code O10–O16), maternal type 2 diabetes mellitus (ICD‐9‐CM code 250, ICD‐10‐CM code E11), and pregnancy‐related complications. Additionally, NHI contributions, which were charged according to monthly income, were collected as a surrogate for the family’s socioeconomic status.

### 2.4. Statistical Analysis

For descriptive statistics, continuous variables were presented by mean and standard deviation. Numbers and percentages were used for categorical data. Independent *t*‐tests were used to compare continuous variables, and chi‐square tests were used to compare categorical data. The association between maternal autoimmune disease and risk of T1DM was examined by both multiple and conditional logistic regression models to adjust for gender, birth weight, gestational age, maternal age, maternal chronic disease, mode of delivery, pregnancy‐related complications, family income, and urbanization. SAS statistical package (version 9.4; SAS Institute, Cary, North Carolina, USA) was employed for all data analysis. A *p*‐value of less than 0.05 was regarded as statistically significant.

## 3. Results

**Figure 1:**
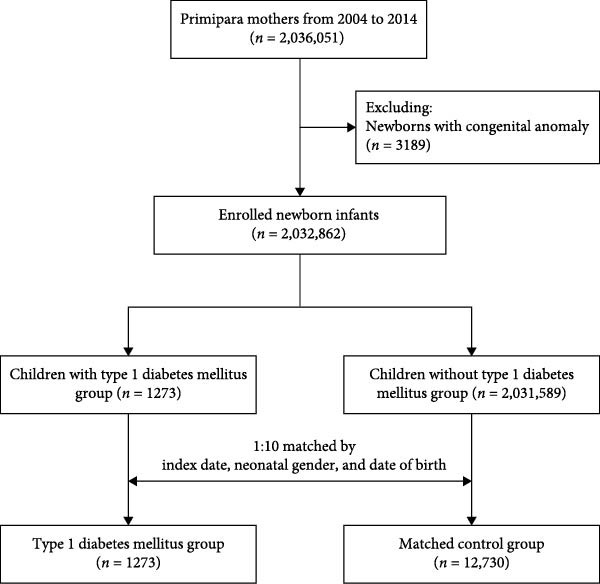
Composition of the study cohort.

This nested case–control study included 2,032,862 newborns in Taiwan between 2004 and 2014, after excluding 3189 infants with congenital anomalies (ICD‐9 codes 740–758) from the initial cohort of 2,036,051 (Figure 1). Among them, 1273 infants were diagnosed with type 1 diabetes (T1DM) and registered in the Catastrophic Illness Registry. A control group of 12,730 infants was randomly selected from the remaining cohort, matched at a 1:10 ratio by birth date, sex, and index date. Follow‐up continued until December 31, 2020. To comprehensively assess the impact of maternal autoimmune diseases, mothers with T1DM prior to delivery were also included. Thus, a total of 347 mothers with autoimmune diseases (including T1DM) were identified in the study population.

### 3.1. Baseline Characteristics of T1DM and the Control Group

Table 1 revealed no significant differences in age, gender, maternal age, urbanization, or most maternal comorbidities between the two groups. However, there was a notable difference in family income distribution (*p* = 0.004), with lower income levels being more prevalent in the T1DM group. Both groups exhibited a similar rate of vaginal deliveries, and factors such as birth weight, gestational age, and pregnancy‐related complications were comparable. We conducted additional demographic analyses stratified by the presence of maternal autoimmune disease (*n* = 347), as shown in Table S1. The results indicated that mothers with autoimmune diseases were older, had higher income, and were at increased risk for preterm birth, hypertension, and diabetes. Infants born to these mothers were also younger at enrollment.

**Table 1:** Baseline characteristics of type 1 diabetes mellitus (T1DM) and control groups.

Characteristics	Non‐T1DM group (n = 12,730)	T1DM group (n = 1273)	*p-Value*
	n (%)	n (%)	
Age (years)			1.00
< 5	3860 (30.3)	386 (30.3)	—
5–9	5690 (44.7)	569 (44.7)	—
≥ 10	3180 (25)	318 (25)	—
Sex			1.00
Female	6980 (54.8)	698 (54.8)	—
Male	5750 (45.2)	575 (45.2)	—
Birth weight (g)			0.24
≥ 2500	11,870 (93.2)	1198 (94.1)	—
< 2500	860 (6.8)	75 (5.9)	—
Maternal age			0.25
< 25	1979 (15.5)	180 (14.1)	—
25–29	4378 (34.4)	470 (36.9)	—
30–34	4572 (35.9)	442 (34.7)	—
≥ 35	1801 (14.1)	181 (14.2)	—
Mode of delivery			0.45
Vaginal delivery	8275 (65)	814 (63.9)	—
Cesarean section	4455 (35)	459 (36.1)	—
Maternal comorbidity			—
Type 2 diabetes mellitus (T2DM)	104 (0.8)	21 (1.6)	0.003
Hypertension	139 (1.1)	12 (0.9)	0.62
Pregnancy‐related complication			—
Gestational diabetes mellitus (GDM)	127 (1)	17 (1.3)	0.26
Placenta previa or abruptio placentae	176 (1.4)	17 (1.3)	0.89
Anemia	67 (0.5)	14 (1.1)	0.010
Gestational age			0.06
≥ 37 weeks	11,759 (92.4)	1157 (90.9)	—
< 37 weeks	971 (7.6)	116 (9.1)	—
Family income			0.004
NT$ ≤ 18780	3612 (28.4)	398 (31.3)	—
NT$18781–27,600	4627 (36.3)	487 (38.3)	—
NT$27601–42,000	2756 (21.6)	250 (19.6)	—
NT$ > 42000	1735 (13.6)	138 (10.8)	—
Maternal autoimmune diseases	290 (2.3)	57 (4.5)	< 0.001
Systemic lupus erythematosus	44 (0.3)	6 (0.5)	0.47
Sjogren’s syndrome	52 (0.4)	10 (0.8)	0.05
Rheumatoid arthritis	28 (0.2)	7 (0.5)	0.025
Hashimoto thyroiditis	29 (0.2)	11 (0.9)	< 0.001
Graves’ disease	109 (0.9)	20 (1.6)	0.011
Systemic sclerosis + Dermatomyositis + Celiac disease + Addison’s disease + Psoriasis	50 (0.4)	3 (0.2)	N/A
T1DM	10 (0.1)	9 (0.7)	< 0.001

Abbreviation: N/A, not applicable due to insufficient case numbers.

### 3.2. Multiple Logistic Regression Analysis of Factors Associated With T1DM

After adjusting for cofactors, the multiple logistic regression model revealed that mothers with autoimmune disease had a higher risk of T1DM (adjusted odds ratio [aOR]: 1.95, 95% CI: 1.45–2.63, *p*  < 0.001). Maternal type 2 diabetes mellitus (aOR: 1.74, 95% CI: 1.06–2.83, *p* = 0.027), preterm birth (aOR: 1.33, 95% CI: 1.06–1.67, *p* = 0.015), and anemia (aOR: 2.09, 95% CI: 1.16–3.75, *p* = 0.014) were positively associated with an increased risk of T1DM in offspring. Conversely, higher family income (above NT$42,000) and low birth weight (< 2500 g) were associated with a reduced risk of T1DM (aOR: 0.70, 95% CI: 0.57–0.87, *p* = 0.001; aOR: 0.72, 95% CI: 0.55–0.95, *p* = 0.020, respectively). (Table 2) No significant associations were observed for other maternal comorbidities or perinatal factors. These findings were further supported by additional analyses using conditional logistic regression models, both unstratified and stratified by age and sex, as detailed in Tables S2 and S3.

**Table 2:** Multiple logistic regression model results for risk of type 1 diabetes mellitus (T1DM).

Variables	Odds ratio	95% CI	*p*‐Value
Maternal autoimmune disease	1.95	1.45−2.63	<0.001 ^∗^
Age (years)	—	—	—
<5	1.00	—	—
5–9	1.00	0.87−1.15	0.99
≥10	1.00	0.85−1.17	0.96
Gender	—	—	—
Female	1.00	—	—
Male	0.99	0.88−1.11	0.83
Birth weight (g)	—	—	—
≥2500	1.00	—	—
<2500	0.72	0.55−0.95	0.020 ^∗^
Gestational age	—	—	—
≥37 weeks	1.00	—	—
<37 weeks	1.33	1.06−1.67	0.015 ^∗^
Mode of delivery	—	—	—
Vaginal delivery	1.00	—	—
Cesarean section	1.03	0.91−1.16	0.70
Maternal age	—	—	—
<25	1.00	—	—
25–29	1.22	1.02−1.46	0.033 ^∗^
30–34	1.13	0.94−1.37	0.20
≥35	1.19	0.95−1.49	0.13
Family income	—	—	—
NT$ ≤ 18780	1.00	—	—
NT$ 18,781–27,600	0.94	0.81−1.08	0.36
NT$ 27,601–42,000	0.80	0.67−0.95	0.010 ^∗^
NT$ > 42000	0.70	0.57−0.87	0.001 ^∗^
Pregnancy‐related complications	—	—	—
Gestational diabetes mellitus (GDM)	1.34	0.80−2.23	0.27
Placenta previa or abruptio	0.90	0.54−1.49	0.68
Anemia	2.09	1.16−3.75	0.014 ^∗^
Maternal comorbidity	—	—	—
Hypertension	0.75	0.41−1.37	0.35
Type 2 diabetes mellitus (T2DM)	1.74	1.06−2.83	0.027 ^∗^

*Note:* Model adjusted for gender, age, birth weight, mode of delivery, gestational age, mother’s age, maternal comorbidities, maternal autoimmune diseases, family income, and pregnancy‐related complications.

Abbreviations: CI, confidence interval; NT$, New Taiwan dollars.

^∗^ *p* < 0.05.

### 3.3. Associations Between Maternal Autoimmune Disorders and Risk of T1DM

The multivariable analysis presented in Table 3 elucidates the associations between various autoimmune diseases and the presence of T1DM after adjusting for age, gender, birth weight, mode of delivery, maternal age, gestational age, maternal comorbidity, pregnancy‐related complications, maternal autoimmune disease, and family income. Maternal T1DM was associated with the highest risk of offspring T1DM (aOR: 6.81, 95% CI: 2.30–20.16, *p*  < 0.001). Significant associations were also observed for Hashimoto thyroiditis (aOR: 3.75, 95% CI: 1.85–7.60, *p*  < 0.001), rheumatoid arthritis (aOR: 2.49, 95% CI: 1.08–5.77, *p* = 0.033), and Graves’ disease (aOR: 1.85, 95% CI: 1.14–2.99, *p* = 0.013). Other maternal autoimmune conditions, such as systemic lupus erythematosus, Sjogren’s syndrome, and systemic sclerosis, did not show statistically significant associations with offspring T1DM. Conditions with sparse case numbers, such as dermatomyositis, celiac disease, Addison’s disease, and psoriasis, were not included in the regression model due to insufficient sample size. To further examine potential genetic contributions, we conducted an additional multivariable model adjusting for paternal T1DM (Table S4). Maternal autoimmune diseases remained significantly associated with offspring T1DM (aOR 1.96, 95% CI 1.46–2.65), while paternal T1DM conferred an even higher risk (aOR 9.58, 95% CI 3.67–25.05). A sensitivity analysis adjusting for paternal T1DM (Table S5) showed consistent associations for Hashimoto thyroiditis, Graves’ disease, and rheumatoid arthritis, supporting the robustness of the primary findings.

**Table 3:** Multivariate analysis of maternal autoimmune disease associated with type 1 diabetes mellitus (T1DM).

Variables	Non‐T1DM group (%)	T1DM group (%)	aOR	95%CI	*p-Value*
Autoimmune diseases	290 (2.3)	57 (4.5)	1.95	1.45−2.63	<0.001 ^∗^
Systemic lupus erythematosus	44 (0.3)	6 (0.5)	1.40	0.59−3.33	0.44
Sjogren’s syndrome	52 (0.4)	10 (0.8)	1.88	0.95−3.73	0.07
Rheumatoid arthritis	28 (0.2)	7 (0.5)	2.49	1.08−5.77	0.033 ^∗^
Hashimoto thyroiditis	29 (0.2)	11 (0.9)	3.75	1.85−7.60	<0.001 ^∗^
Graves’ disease	109 (0.9)	20 (1.6)	1.85	1.14−2.99	0.013 ^∗^
Systemic sclerosis	50(0.4)	3 (0.2)	3.50	0.36−34.08	0.28
Dermatomyositis			N/A	N/A	N/A
Celiac disease			N/A	N/A	N/A
Addison’s disease			N/A	N/A	N/A
Psoriasis			N/A	N/A	N/A
T1DM	10 (0.1)	9 (0.7)	6.81	2.30−20.16	<0.001 ^∗^

*Note:* Model adjusted for gender, age, birth weight, mode of delivery, gestational age, mother’s age, maternal comorbidities, maternal autoimmune diseases, family income, and pregnancy‐related complications.

Abbreviations: aOR, adjusted odds ratio; CI, confidence interval; N/A, not applicable.

^∗^ *p* < 0.05.

## 4. Discussion

In this nationwide cohort study with long‐term follow‐up, we explored the potential connection between maternal autoimmune diseases and the risk of childhood T1DM. Our findings demonstrate that certain maternal autoimmune conditions, particularly autoimmune thyroiditis and rheumatoid arthritis, are associated with an increased risk of T1DM in offspring. These results contribute valuable evidence to the understanding of intergenerational autoimmune disease relationships.

In Taiwan, individuals with a family history of Sjogren’s syndrome have been reported to be associated with an increased risk of developing T1DM [10]. However, another previously published study in Taiwan did not identify a significant correlation between T1DM and rheumatoid arthritis in subgroup analysis. There are two likely explanations for this discrepancy. First, there was a significant portion of loss to follow‐up, with nearly half of the cases lost before 6 years of age. Second, the study employed a traditional cohort design, which can result in limited statistical power when investigating rare diseases [11]. In contrast, our study’s comprehensive design and robust follow‐up strengthen the evidence for an association between maternal autoimmunity and offspring’s T1DM risk.

Several other factors have been identified as potential contributors to T1DM in children, including higher birth weight, Cesarean delivery, maternal obesity during pregnancy, preconception parental obesity, enterovirus infections, advanced maternal age, and maternal gluten consumption [1, 12]. Furthermore, maternal adversity, such as asthma during pregnancy, has been shown through epidemiological studies and genome‐wide DNA methylation analyses to induce persistent and widespread changes in fetal epigenetic patterns [13–15]. These alterations influence genes involved in innate and adaptive immunity, along with those implicated in T1DM [16]. For example, studies on monozygotic twins discordant for T1DM revealed distinct epigenetic variations in CD14+ monocytes [15]. While the association between maternal antibody‐mediated diseases and antibody transmission, like thyroiditis and systemic lupus erythematosus, is well‐established [17, 18], the role of maternal T1DM autoantibodies remains contentious. Some research suggests that exposure to insulin autoantibodies (IAA) does not increase diabetes risk in non‐obese diabetic (NOD) mice [19], while other studies report a higher prevalence of beta cell‐specific autoantibodies in the umbilical cord blood of offspring who later develop T1DM [20]. These findings suggest that maternal autoantibodies might act as a risk factor, but further research is needed to confirm this hypothesis.

Genetic factors also play a critical role in the heritability of T1DM. Genetic susceptibility loci for T1DM often overlap with those for other autoimmune diseases that frequently cluster within families affected by T1DM [21, 22]. Our study supports these observations, as we found that mothers with autoimmune thyroiditis were more likely to have children with T1DM. Both T1DM and autoimmune thyroiditis, which includes Hashimoto’s thyroiditis and Graves’ disease, are chronic autoimmune conditions that commonly co‐occur due to shared immunogenetic susceptibilities [23, 24]. T1DM primarily targets insulin‐producing β‐cells in the pancreas, whereas autoimmune thyroiditis affects thyroid tissue, causing hypothyroidism or hyperthyroidism. These diseases share key immunological features, including the production of organ‐specific autoantibodies—such as anti‐thyroid peroxidase antibodies (TPOAb) in autoimmune thyroiditis and glutamic acid decarboxylase antibodies (GADAb) in T1DM—and infiltration of T and B lymphocytes into affected organs [25]. The shared genetic predisposition for these diseases is particularly evident in the human leukocyte antigen (HLA) system, with haplotypes like DR3‐DQ2 and DR4‐DQ8 linked to susceptibility in both conditions [26]. Additionally, non‐HLA genes such as CTLA‐4 and PTPN22 underscore the genetic overlap, influencing immune regulation and autoantigen presentation [27, 28]. Clinical studies indicate that up to 25% of individuals with T1DM develop thyroid autoantibodies, with about 10% progressing to overt autoimmune thyroiditis [29]. These shared mechanisms highlight the potential for developing targeted therapies that address common pathways, potentially reducing the burden of these autoimmune diseases. Teplizumab, an anti‐CD3 monoclonal antibody, has shown potential in delaying the onset of T1DM in at‐risk individuals by modulating T‐cell activity and preserving pancreatic β‐cell function [30, 31]. Future studies could explore its benefits for children predisposed to T1DM due to maternal autoimmune conditions, further advancing therapies targeting shared pathways in autoimmune diseases.

Based on the earlier investigation, T1DM and rheumatoid arthritis also showed a familial genetic predisposition and a shared mechanism, which involves processes like lysosome and Fc gamma R‐mediated phagocytosis [32]. Genetic or immunological links could be present among autoimmune thyroiditis, rheumatoid arthritis, and T1DM. Additional research is required to determine how prenatal events and maternal comorbidities may influence the development of childhood‐onset T1DM.

This study has several limitations. First, our data were derived from NHI claims, which did not include laboratory findings, such as genetic data or detailed immunological profiles. Second, disease diagnoses relied on physician coding, which may have introduced some degree of misclassification. Third, the study lacked comprehensive information on maternal nutritional intake, physical activity, hygiene, and environmental exposures during pregnancy. While we attempted to mitigate confounding factors by adjusting for pregnancy‐related complications, maternal comorbidities, and perinatal conditions in neonates, residual confounding may still exist. Our current analysis focused specifically on T1DM outcomes and did not explore the broader risk of other autoimmune diseases in children born to affected versus unaffected mothers. However, as the maximum follow‐up period in our study was 14 years, most of the enrolled infants had not yet reached adolescence, a period when many autoimmune diseases other than T1DM commonly emerge. As such, the incidence of these conditions may have been substantially underestimated, and we therefore chose to concentrate this analysis on T1DM. The low prevalence of celiac disease in this cohort, resulting in its exclusion from regression analyses, likely reflects its rarity in East Asian populations due to limited genetic susceptibility and low gluten consumption, further complicated by possible underdiagnosis in administrative data reliant on ICD codes without serological confirmation [33]. The absence of maternal outpatient visit data may limit insights into healthcare‐seeking patterns; however, its impact is likely minimal given Taiwan’s universal health coverage and the focus on established covariates.

## 5. Conclusions

Maternal autoimmune conditions, especially T1DM, autoimmune thyroiditis, and rheumatoid arthritis, might heighten the likelihood of offspring developing T1DM. These findings underscore the importance of targeted screening programs for T1DM in children of affected mothers, such as early autoantibody testing or genetic risk assessment, to enable timely intervention and potentially delay onset. Further prospective long‐term follow‐up studies are needed to elucidate the causal relationships.

## Disclosure

Ching‐Heng Lin takes responsibility for the integrity of the data and the accuracy of the data analysis.

## Conflicts of Interest

The authors declare no conflicts of interest.

## Author Contributions

Hsin‐Chien Yen wrote the manuscript. Ching‐Heng Lin performed the data analysis. Ming‐Chih Lin designed the research and reviewed the final version of the manuscript.

## Funding

This study was supported by Taichung Veterans General Hospital Research Fund (Registration Numbers TCVGH‐1136501C, 1136503C, 1136501B, 1136510B, PU1138103, FCU 1138204).

## Supporting Information

Additional supporting information can be found online in the Supporting Information section.

## Supporting information


**Supporting Information** Table S1. Baseline characteristics of participants stratified by maternal autoimmune disease status. Table S2. Conditional logistic regression of maternal autoimmune diseases and risk of pediatric type 1 diabetes mellitus (T1DM), unstratified analysis. Table S3. Conditional logistic regression of maternal autoimmune diseases and risk of pediatric type 1 diabetes mellitus (T1DM), stratified by age and sex. Table S4. Multiple logistic regression model results for risk of type 1 diabetes mellitus (T1DM). Table S5. Multivariate analysis of maternal autoimmune disease associated with type 1 diabetes mellitus (T1DM).

## Data Availability

To protect patient confidentiality and ensure the reliability of the databases, investigators are required to conduct onsite analyses at the Health and Welfare Data Science Center in Taiwan via a remote connection to the Ministry of Health and Welfare servers. Dr. Ching‐Heng Lin (epid@vghtc.gov.tw) had full access to all the data in the study.
