# Supplementary material for: Maternal Autoimmune Disease and Childhood‐Onset Type 1 Diabetes: A Nationwide Population‐Based Nested Case‐Control Study
Source: Pediatr Diabetes. 2026 Jan 20;2026:3418021. doi: 10.1155/pedi/3418021 (PMC12820419; doi:10.1155/pedi/3418021)
Supplement: Supplementary file 1 — Supporting Information Table S1. Baseline characteristics of participants stratified by maternal autoimmune disease status. Table S2. Conditional logistic regression of maternal autoimmune diseases and risk of pediatric type 1 diabetes mellitus (T1DM), unstratified analysis. Table S3. Conditional logistic regression of maternal autoimmune diseases and risk of pediatric type 1 diabetes mellitus (T1DM), stratified by age and sex. Table S4. Multiple logistic regression model results for risk of type 1 diabetes mellitus (T1DM). Table S5. Multivariate analysis of maternal autoimmune disease associated with type 1 diabetes mellitus (T1DM). [file PEDI-2026-3418021-s001.docx]

**Table S1. Baseline characteristics of participants stratified by maternal autoimmune disease status.**

| **Characteristics** | **Mothers without autoimmune diseases (n=13,656)** | | **Mothers with autoimmune diseases**  **(n=347)** | ***p-value*** |
| --- | --- | --- | --- | --- |
|  | **n(%)** | **n (%)** | |  |
| **Age (years)** |  |  | | <0.001 |
| <5 | 4103 (30.0) | 143 (41.2) | |  |
| 5-9 | 6122 (44.8) | 137 (39.5) | |  |
| **≥**10 | 3431 (25.1) | 67 (19.3) | |  |
| **Gender** |  |  | | 0.22 |
| Female | 7499 (54.9) | 179 (51.6) | |  |
| Male | 6157 (45.1) | 168 (48.4) | |  |
| **Birth weight (g)** |  |  | | 0.29 |
| ≥2500 | 12749 (93.4) | 319 (91.9) | |  |
| <2500 | 907 (6.6) | 28 (8.1) | |  |
| **Gestational age** |  |  | | 0.025 |
| ≥37wks | 12607 (92.3) | 309 (89.0) | |  |
| <37wks | 1049 (7.7) | 38 (11.0) | |  |
| **Mode of delivery** |  |  | | 0.29 |
| Vaginal delivery | 8873 (65) | 216 (62.2) | |  |
| Cesarean section | 4783 (35) | 131 (37.8) | |  |
| **Maternal age** |  |  | | <0.001 |
| <25 | 2134 (15.6) | 25 (7.2) | |  |
| 25-29 | 4749 (34.8) | 99 (28.5) | |  |
| 30-34 | 4849 (35.5) | 165 (47.6) | |  |
| **≥**35 | 1924 (14.1) | 58 (16.7) | |  |
| **Family income** |  |  | | 0.003 |
| NT$ ≤18780 | 3931 (28.8) | 79 (22.8) | |  |
| NT$ 18781-27600 | 4988 (36.5) | 126 (36.3) | |  |
| NT$ 27601-42000 | 2931 (21.5) | 75 (21.6) | |  |
| NT$ >42000 | 1806 (13.2) | 67 (19.3) | |  |
| **Pregnancy-related complications** |  |  | |  |
| Gestational diabetes mellitus (GDM) | 140 (1.0) | 4 (1.2) | | 0.82 |
| Placenta previa or abruptio | 187 (1.4) | 8 | | 0.57 |
| Anemia | 79 (0.6) |  |  | 1.00 |
| **Maternal comorbidity** |  |  | |  |
| Hypertension | 139 (1.0) | 12 (3.5) | | <0.001 |
| Type 2 diabetes mellitus (T2DM) | 99 (0.7) | 26 (7.5) | | <0.001 |

**Table S2. Conditional logistic regression of maternal autoimmune diseases and risk of pediatric type 1 diabetes mellitus (T1DM), unstratified analysis.^a^**

| **Variables** | **aOR** | **95% CI** | ***p*-value** |
| --- | --- | --- | --- |
| **Maternal autoimmune disease** | 1.95 | 1.45-2.63 | <0.001 |
| **Birth weight (g)** |  |  |  |
| ≥2500 | 1.00 |  |  |
| <2500 | 0.72 | 0.55-0.95 | 0.019 |
| **Gestational age** |  |  |  |
| ≥37wks | 1.00 |  |  |
| <37wks | 1.33 | 1.06-1.67 | 0.015 |
| **Mode of delivery** |  |  |  |
| Vaginal delivery | 1.00 |  |  |
| Cesarean section | 1.03 | 0.91-1.16 | 0.68 |
| **Maternal age** |  |  |  |
| <25 | 1.00 |  |  |
| 25-29 | 1.22 | 1.02-1.46 | 0.033 |
| 30-34 | 1.13 | 0.94-1.37 | 0.20 |
| **≥**35 | 1.19 | 0.95-1.49 | 0.14 |
| **Family income** |  |  |  |
| NT$ ≤18780 | 1.00 |  |  |
| NT$ 18781-27600 | 0.94 | 0.81-1.08 | 0.36 |
| NT$ 27601-42000 | 0.80 | 0.67-0.95 | 0.010 |
| NT$ >42000 | 0.70 | 0.57-0.87 | 0.001 |
| **Pregnancy-related complications** |  |  |  |
| Gestational diabetes mellitus (GDM) | 1.34 | 0.80-2.23 | 0.27 |
| Placenta previa or abruptio | 0.90 | 0.54-1.50 | 0.69 |
| Anemia | 2.07 | 1.15-3.72 | 0.015 |
| **Maternal comorbidity** |  |  |  |
| Hypertension | 0.74 | 0.41-1.36 | 0.34 |
| Type 2 Diabetes mellitus (T2DM) | 1.73 | 1.06-2.82 | 0.028 |

^a^ Adjusted odds ratios (aORs) for maternal autoimmune diseases, controlling for birth weight, gestational age, mode of delivery, maternal age, family income, pregnancy-related complications, and maternal comorbidities. Age and sex were controlled through 1:10 matching. CI: confidence interval. NT$: New Taiwan dollars

**Table S3. Conditional logistic regression of maternal autoimmune diseases and risk of pediatric type 1 diabetes mellitus (T1DM), stratified by age and sex.^b^**

| **Variables** | **aOR** | **95% CI** | ***p*-value** |
| --- | --- | --- | --- |
| **Maternal autoimmune disease** | 1.95 | 1.45-2.62 | <0.001 |
| **Age (years)** |  |  |  |
| <5 | 1.00 |  |  |
| 5-9 | 1.00 | 0.87-1.15 | 0.99 |
| **≥**10 | 1.00 | 0.85-1.17 | 0.96 |
| **Sex** |  |  |  |
| Female | 1.00 |  |  |
| Male | 0.99 | 0.88-1.11 | 0.83 |
| **Birth weight (g)** |  |  |  |
| ≥2500 | 1.00 |  |  |
| <2500 | 0.72 | 0.55-0.95 | 0.020 |
| **Gestational age** |  |  |  |
| ≥37wks | 1.00 |  |  |
| <37wks | 1.33 | 1.06-1.67 | 0.015 |
| **Mode of delivery** |  |  |  |
| Vaginal delivery | 1.00 |  |  |
| Cesarean section | 1.03 | 0.91-1.16 | 0.70 |
| **Maternal age** |  |  |  |
| <25 | 1.00 |  |  |
| 25-29 | 1.22 | 1.02-1.46 | 0.033 |
| 30-34 | 1.13 | 0.94-1.37 | 0.20 |
| **≥**35 | 1.19 | 0.95-1.49 | 0.13 |
| **Family income** |  |  |  |
| NT$ ≤18780 | 1.00 |  |  |
| NT$ 18781-27600 | 0.94 | 0.81-1.08 | 0.36 |
| NT$ 27601-42000 | 0.80 | 0.67-0.95 | 0.010 |
| NT$ >42000 | 0.70 | 0.57-0.87 | 0.001 |
| **Pregnancy-related complications** |  |  |  |
| Gestational diabetes mellitus (GDM) | 1.34 | 0.80-2.23 | 0.27 |
| Placenta previa or abruptio | 0.90 | 0.54-1.49 | 0.68 |
| Anemia | 2.09 | 1.16-3.75 | 0.014 |
| **Maternal comorbidity** |  |  |  |
| Hypertension | 0.75 | 0.41-1.37 | 0.35 |
| Type 2 Diabetes mellitus (T2DM) | 1.74 | 1.06-2.83 | 0.027 |

^b^ Model adjusted for birth weight, mode of delivery, gestational age, maternal age, family income, pregnancy-related complications and maternal comorbidities. Stratified models present adjusted odds ratios (aORs) for age groups (<5, 5-9, ≥10 years) and sex (male, female). CI: confidence interval. NT$: New Taiwan dollars

**Table S4. Multiple logistic regression model results for risk of type 1 diabetes mellitus (T1DM).^b.c^**

| **Variables** | **Odds ratio** | **95% CI** | ***p*-value** |
| --- | --- | --- | --- |
| **Maternal autoimmune disease** | 1.96 | 1.46-2.65 | <0.001 |
| **Paternal comorbidity** |  |  |  |
| Type 1 diabetes mellitus (T1DM) | 9.58 | 3.67-25.05 | <0.001 |
| **Age (years)** |  |  |  |
| <5 | 1.00 |  |  |
| 5-9 | 1.00 | 0.87-1.15 | 0.991 |
| **≥**10 | 0.96 | 0.82-1.13 | 0.632 |
| **Gender** |  |  |  |
| Female | 1.00 |  |  |
| Male | 0.99 | 0.87-1.11 | 0.803 |
| **Birth weight (g)** |  |  |  |
| ≥2500 | 1.00 |  |  |
| <2500 | 0.71 | 0.54-0.95 | 0.021 |
| **Gestational age** |  |  |  |
| ≥37wks | 1.00 |  |  |
| <37wks | 1.33 | 1.05-1.68 | 0.020 |
| **Mode of delivery** |  |  |  |
| Vaginal delivery | 1.00 |  |  |
| Cesarean section | 1.00 | 0.88-1.14 | 0.982 |
| **Maternal age** |  |  |  |
| <25 | 1.00 |  |  |
| 25-29 | 1.20 | 0.99-1.44 | 0.062 |
| 30-34 | 1.10 | 0.90-1.33 | 0.353 |
| **≥**35 | 1.19 | 0.94-1.50 | 0.148 |
| **Family income** |  |  |  |
| NT$ ≤18780 | 1.00 |  |  |
| NT$ 18781-27600 | 0.96 | 0.83-1.11 | 0.586 |
| NT$ 27601-42000 | 0.82 | 0.69-0.98 | 0.025 |
| NT$ >42000 | 0.72 | 0.58-0.89 | 0.002 |
| **Pregnancy-related complications** |  |  |  |
| Gestational diabetes mellitus (GDM) | 1.32 | 0.79-2.21 | 0.295 |
| Placenta previa or abruptio | 0.91 | 0.54-1.53 | 0.716 |
| Anemia | 2.01 | 1.10-3.68 | 0.024 |
| **Maternal comorbidity** |  |  |  |
| Hypertension | 0.71 | 0.37-1.38 | 0.317 |
| Type 2 diabetes mellitus (T2DM) | 1.74 | 1.05-2.87 | 0.031 |

^b^Model adjusted for gender, age, birth weight, mode of delivery, gestational age, mother’s age, maternal comorbidities, maternal autoimmune diseases, family income, pregnancy-related complications and paternal type 1 diabetes mellitus. CI: confidence interval. NT$: New Taiwan dollars

^c^The cohort includes 13,450 mother-child pairs (Non-T1D Group: 12,226; T1D Group: 1,224) due to 4% missing paternal data.

**Table S5. Multivariate analysis of maternal autoimmune disease associated with type 1 diabetes mellitus (T1DM).^d^**

| **Variables** | **aOR** | **95%CI** | ***P-value*** |
| --- | --- | --- | --- |
| **Autoimmune diseases** | 1.96 | 1.45-2.64 | <0.001 |
| Systemic lupus erythematosus | 1.39 | 0.59-3.32 | 0.452 |
| Sjogren's syndrome | 1.90 | 0.96-3.79 | 0.067 |
| Rheumatoid arthritis | 2.58 | 1.11-5.98 | 0.028 |
| Hashimoto thyroiditis | 3.38 | 1.63-7.02 | 0.001 |
| Graves' disease | 1.91 | 1.17-3.10 | 0.009 |
| Systemic sclerosis | 3.69 | 0.38-36.22 | 0.263 |
| Dermatomyositis | N/A | N/A | N/A |
| Celiac disease | N/A | N/A | N/A |
| Addison's disease | N/A | N/A | N/A |
| Psoriasis | N/A | N/A | N/A |
| T1D | 7.29 | 2.42-22.03 | <0.001 |

^d^Model adjusted for gender, age, birth weight, mode of delivery, gestational age, mother’s age, maternal comorbidities, maternal autoimmune diseases, family income, pregnancy-related complications and paternal type 1 diabetes mellitus.

aOR: adjusted odds ratio; CI: confidence interval; N/A: excluded due to insufficient sample size
